# Antimicrobial Potential of Strontium‐Functionalized Titanium Against Bacteria Associated With Peri‐Implantitis

**DOI:** 10.1002/cre2.903

**Published:** 2024-07-19

**Authors:** Hatem Alshammari, Jessica Neilands, Christian Sloth Jeppesen, Klaus Pagh Almtoft, Ole Zoffmann Andersen, Andreas Stavropoulos

**Affiliations:** ^1^ Department of Preventive Dentistry, College of Dentistry University of Hail Hail Saudi Arabia; ^2^ Periodontology, Faculty of Odontology University of Malmö Malmö Sweden; ^3^ Department of Oral Biology, Faculty of Odontology University of Malmö Malmö Sweden; ^4^ Tribology Center Danish Technological Institute Aarhus Denmark; ^5^ Department of Periodontology University of Bern Bern Switzerland; ^6^ Institute Straumann AG Basel Switzerland; ^7^ Division of Conservative Dentistry and Periodontology University Clinic of Dentistry, Medical University of Vienna Vienna Austria; ^8^ Department of Periodontology Blekinge Hospital Karlskrona Sweden

**Keywords:** antimicrobial coatings, dental implant, peri‐implantitis, strontium, titanium

## Abstract

**Objectives:**

To explore the antimicrobial potential of strontium (Sr)‐functionalized wafers against multiple bacteria associated with per‐implant infections, in both mono‐ and multispecies biofilms.

**Materials and Methods:**

The bactericidal and bacteriostatic effect of silicon wafers functionalized with a strontium titanium oxygen coating (Sr‐Ti‐O) or covered only with Ti (controls) against several bacteria, either grown as a mono‐species or multispecies biofilms, was assessed using a bacterial viability assay and a plate counting method. Mono‐species biofilms were assessed after 2 and 24 h, while the antimicrobial effect on multispecies biofilms was assessed at Days 1, 3, and 6. The impact of Sr functionalization on the total percentage of *Porphyromonas gingivalis* in the multispecies biofilm, using qPCR, and gingipain activity was also assessed.

**Results:**

Sr‐functionalized wafers, compared to controls, were associated with statistically significant less viable cells in both mono‐ and multispecies tests. The number of colony forming units (CFUs) within the biofilm was significantly less in Sr‐functionalized wafers, compared to control wafers, for *Staphylococcus aureus* at all time points of evaluation and for *Escherichia coli* at Day 1. Gingipain activity was less in Sr‐functionalized wafers, compared to control wafers, and the qPCR showed that *P. gingivalis* remained below detection levels at Sr‐functionalized wafers, while it consisted of 15% of the total biofilm on control wafers at Day 6.

**Conclusion:**

Sr functionalization displayed promising antimicrobial potential, possessing bactericidal and bacteriostatic ability against bacteria associated with peri‐implantitis grown either as mono‐species or mixed in a multispecies consortium with several common oral microorganisms.

## 1 Introduction

Modern dental implant technology and current surgical techniques have resulted in high osseointegration rates of titanium (Ti) implants, as evident from the very low early implant loss rates reported in several studies (e.g., Jemt 2017; Lin et al. 2018). High implant survival and success rates have also been reported in the long term in patients with a history of periodontal disease (Pandolfi et al. 2019). Nevertheless, bacterial colonization and biofilm formation on the surface of implant reconstructions may cause inflammation in the peri‐implant mucosa (i.e., peri‐implant mucositis) and eventually lead to peri‐implant bone loss (i.e., peri‐implantitis) (Heitz‐Mayfield and Salvi 2018; Klinge et al. 2018). Peri‐implantitis prevalence estimates vary largely depending on the cut‐off value of bone loss, for case definition; for instance, the weighted mean prevalence of peri‐implantitis with ≥ 2 mm bone loss has been estimated to be 22% at the patient level (Derks et al. 2016). Furthermore, peri‐implantitis is often overseen/neglected and patients referred to specialist treatment often show advanced bone destruction and complicated bone defect morphology. Peri‐implantitis treatment is demanding and often involves complex surgery (Donos et al. 2023; Stavropoulos et al. 2019) and is thus associated with substantial patient suffering and high costs; in addition, systemic antibiotics are often prescribed, although not necessarily effective, contributing thereby to the burden of antibiotic resistance. Importantly, recurrence rates in the short to medium term are high, which leads to implant loss (Karlsson et al. 2023). As peri‐implantitis appears to affect a considerable portion of patients with implants and its treatment is challenging, it is important to find effective preventive measures.

On the basis of the understanding that a major component in peri‐implant biological complications is the oral biofilm, preventive strategies have aimed at providing Ti surfaces with antimicrobial potential (Chouirfa et al. 2019; Grischke, Eberhard, and Stiesch 2016); one approach has been combining Ti with other metals. For example, a good antimicrobial effect on various bacteria linked to peri‐implantitis has been shown when silver (Ag) was used, in both in vitro (Choi et al. 2019) and in vivo studies (Masamoto et al. 2021); yet, there are major concerns with this approach due to Ag‐related cytotoxicity (Hadrup, Sharma, and Loeschner 2018; Zhang et al. 2014). Recently, there is increasing interest in strontium (Sr), an alkaline earth metal previously used in the form of strontium ranelate for the treatment of osteoporosis (O'Donnell et al. 2006; Stevenson et al. 2007), because preliminary in vitro studies have indicated that Sr exhibits relevant antibacterial properties (Alshammari, Bakitian, et al. 2021; Alshammari, Neilands, et al. 2021). For example, in an exploratory study, 10 mM of Sr(OH)_2_ achieved significant growth inhibition in planktonic cultures of *Streptococcus mitis*, *Streptococcus epidermidis*, *Aggregatibacter actinomycetemcomitans*, *Escherichia coli*, and *Porphyromonas gingivalis*, and also exhibited strong bactericidal effect in mono‐species biofilm viability assays (Alshammari, Neilands, et al. 2021). Sr is particularly interesting in this context because Sr‐functionalized Ti implants have exhibited enhanced osseointegration in several preclinical in vivo studies (Andersen et al. 2013; López‐Valverde et al. 2019; Offermanns, Andersen, et al. 2018).

In perspective, since peri‐implantitis involves a dysbiotic multimicrobial biofilm attached to a Ti surface, it is relevant to assess the possible antimicrobial potential of Ti surfaces functionalized with Sr against multispecies consortia. Thus, this study assessed the antimicrobial potential of Sr‐functionalized wafers, using magnetron‐sputtering, against bacteria associated with peri‐implantitis in mono‐ and multispecies cultures.

## 2 Materials and Methods

### 2.1 Sample Preparation

Silicon wafers were functionalized using a magnetron‐sputtering process with a target comprising 50% commercially pure Ti and 50% Strontium titanate (SrTiO_3_) to produce a Ti‐Sr‐O surface coating (0.8–2 μm thick); a commercially pure Ti target was used to produce wafers coated with Ti (50 nm thick) as controls (Andersen et al. 2013; Offermanns, Steinmassl, et al. 2018). The wafers were initially cut into small square‐shaped samples, approximately 10 × 10 mm. Before each test, both Sr‐functionalized and Ti control wafers were cleaned using 70% alcohol for 30 s and then rinsed using phosphate‐buffered saline (PBS). Then they were transferred to 12‐well plates (Ibidi μ‐Slide, Ibidi GmbH, Martinsried, Germany) using a sterile tweezer and placed in the testing wells facing upwards.

### 2.2 Bacterial Strains

The following bacterial strains were used: *E. coli* (ATCC 25922), *Staphylococcus aureus* (ATCC29213), as well as clinical strains *Streptococcus oralis* (2009‐213A1), *Actinomyces naeslundii* (BJJ), *Parvimonas micra* (EME), *P. gingivalis* (SUB1), and *Fusobacterium nucleatum* (FMD); all had been recovered from patients with established periodontitis. The bacteria had been identified at the species level as described previously using a combination of colony morphology, appearance after Gram staining, and biochemical or molecular tests (Neilands, Bikker, and Kinnby 2016). Two different modes of culturing were used for testing the antimicrobial effect of the Sr‐functionalized and Ti control wafers, that is, mono‐ and multispecies biofilms.

### 2.3 Mono‐Species Biofilms

Three bacteria, commonly associated with peri‐implantitis, were selected for mono‐species biofilms: *E. coli, S. aureus,* and *P. gingivalis*. Each bacterial isolate was stored at −80°C and recovered on Brucella agars before experimental use. Colony forming units (CFUs) from each strain were vortexed, separately, in either Tryptone yeast extract (TYE) (Becton, Dickinson and Co., Albertslund, Denmark) for *E. coli*, and *S. aureus*, or Brain heart infusion broth (BHI) (Neogen Corporation, Lancashire BL9 7JJ, United Kingdom) for *P. gingivalis* until reaching optical density = 0.1 at OD 600 nm. Afterward, bacterial suspensions were incubated, for aerobic bacteria at 37°C in 5% CO_2_ and for anaerobic bacteria at 37°C 10% H_2_, 5% CO_2_ in N_2_ anaerobically, until reaching log‐phase (approximately reaching optical density = 0.5 at OD 600 nm). Subsequently, 3.5 mL of each bacterial suspension was added to the wafer samples in the 12‐well plates and incubated, as described above, over a rocking platform. To evaluate early bacteria adherence and viability, two time points were set for evaluation: 2 and 24 h. All tests were done in triplicates for each species and time point.

### 2.4 Multispecies Biofilms

Each of the previous bacterial strains (i.e., *E. coli, S. aureus,* and *P. gingivalis*) was seperately mixed with four common oral bacterial species, representing a multispecies oral biofilm; *S. oralis, P. micra, A. naeslundii,* and *F. nucleatum*, giving a mixture of a five species consortia in each test; *P. gingivalis*‐, *S. aureus*‐ and *E. coli*‐multispecies tests*.* As described previously, bacterial strains were stored at −80°C and recovered on Brucella agar before experimental use. A solution of 20% horse serum (Håtunalab AB, Håtunaholm, Sweden) and Fastidious anaerobic broth (FAB) (Neogen Corporation, Lancashire, United Kingdom) at a ratio of 50/50% was prepared and pre‐reduced 24 h before the start of each experiment. Afterward, CFUs from each bacterial strain were mixed into the solution until reaching optical density = 0.1 at OD 600 nm. Two Sr‐functionalized and two Ti control wafers were then placed in 12‐well plates (facing upwards) corresponding to each time point of evaluation (1, 3, and 6 days). The wells were then filled with 3.5 mL of the multispecies suspension prepared earlier, where each multispecies consortium was tested separately and incubated in an anaerobic chamber for 6 h. Following 6 h of incubation, the wafer samples were washed carefully in PBS, then transferred into a new pre‐reduced solution and incubated anaerobically. The tests were done in triplicates for each multispecies consortium.

### 2.5 Bacterial Viability Assay

For each time point of evaluation, for both mono‐species and multispecies cultures, samples of Sr‐functionalized and Ti control wafers were taken from the 12‐well plates and washed carefully with PBS. Afterward, 60 μL of LIVE/DEAD BacLight (Invitrogen, Eugene, USA) was added to the surface of the wafers. After 15 min, the samples were transferred to a 2‐well μ‐Slide (Ibidi μ‐Slide, Ibidi GmbH, Martinsried, Germany) and evaluated under a confocal scanning laser microscope (CSLM, Eclipse TE2000 inverted CSLM, Nikon, Japan). From each sample, a total of 10 CSLM images were taken using the software EZ‐C1 v.3.40 build 691 (Nikon) at a resolution of 512 × 512 pixels and with a zoom factor of 1.0, giving a final pixel resolution of 0.42 μm/pixel. The number of viable cells was analyzed using the software bioImage_L.

### 2.6 Vortexing and Bacterial Cell Detachment

To ensure complete biofilm detachment for plating without affecting bacterial viability, different vortexing times were evaluated for the least amount of time required for complete cell detachment. Evaluation of samples under CSLM showed that all bacterial cells were completely detached from the surface after 20 s, while with vortexing below 20 s, bacterial cells attached to the wafers could still be observed. Thus, vortexing for 20 s was set for cell detachment for both plate‐counting and qPCR.

### 2.7 Plate Counting

Additional Sr‐functionalized and Ti control wafer samples from *E. coli* and *S. aureus* multispecies tests were separately vortexed for 20 s in a pre‐reduced dilution blank. Then, the detached bacterial solution was further diluted, to the 10th, in different dilution blanks, four times (−1, −2, −3, and −4). Subsequently, 200 µL aliquot of each solution was inoculated onto MacConkey plates, for *E. coli* identification, and 110‐plates, for *S. aureus* identification. All plates were then incubated aerobically for 2 days, followed by counting CFUs in each sample.

### 2.8 Proteolytic Activity

Gingipain activity was analyzed utilizing the synthetic fluorogenic substrate BIKKAM‐16 (PepScan Presto B.V., Lelystad, The Netherlands). From the supernatant of *P. gingivalis* multispecies biofilm, 50 µL of the suspension was added to 1.5 µL of BIKKAM‐16 in a 96‐well plate (NUNC, Thermo Fisher Scientific, Roskilde, Denmark). Afterward, fluorescence was measured at 1‐min intervals for 10 min in a Clariostar program, a Fluostar Optima plate reader (BMG Labtech, Offenburg, Germany).

### 2.9 qPCR

Total CFUs and the percentage of *P. gingivalis* attached to Sr‐functionalized and Ti control wafers were assessed using a Quantstudio 3 Real‐Time PCR system (Thermo Fisher Scientific). DNA was extracted using a QIAamp UCP Pathogen mini‐kit (Qiagen) according to the manufacturer's instructions, with additional lysozyme treatment step (20 mg/mL lysozyme in 20 mM Tris pH 8.0, 2 mM EDTA, 1,2% Triton X‐100), 37°C for 1 h). The specific primer sequences used for the detection of *P. gingivalis* were: 5′‐TGTAGATGACTGATGGTGAAAACC‐3′ and 5′‐ACGTCATCCCCACCTTCCTC‐3′.

Each primer was diluted from 20 pmol/μL to 5 μM in ultrapure water and kept at −20°C until use. Each reaction mixture (final volume of 10 μL) contained 1 μL DNA template, 0.5 μM forward and reverse primer, 5 μL Power SYBR Green Master Mix (Thermo Fisher Scientific), and 2 μL nuclease‐free ultrapure water. All reactions were done in triplicates and with negative controls. The thermocycling protocol used an initial step of 2 min at 50°C followed by 10 min at 95°C, and 40 cycles of 15 s at 95°C and 1 min at 60°C thereafter. Reaction specificities were confirmed by melting curve analysis. Estimates of cell number were made against standards prepared from 10‐fold serial dilutions of each bacteria (ranging from 65 ng/μL to 0.65 pg/μL) and quantifications were made based on the estimated 16S rRNA gene copy numbers and amounts of chromosomal DNA in each *P. gingivalis* cell (four copies, 2.5 fg).

### 2.10 Statistical Analysis

All experiments were done in triplicates and statistical analysis was performed using SPSS 27 (IBM Corp. Released 2020, IBM SPSS Statistics for Macintosh, Version 27.0. Armonk, NY: IBM Corp). The Mann–Whitney *U* test was used to assess differences between Sr‐functionalized and Ti control wafers in the number of viable cells, in both mono‐ and multispecies cultures (bacterial viability assay) and differences in mean CFUs in multispecies cultures (plate counting). The differences were regarded as significant if *p* < 0.05 and graphically presented as: *p < 0.05, **p < 0.01, ***p < 0.001.

## 3 Results

### 3.1 Effect of Sr on Biofilm Growth and Bacterial Viability

**Figure 1:**
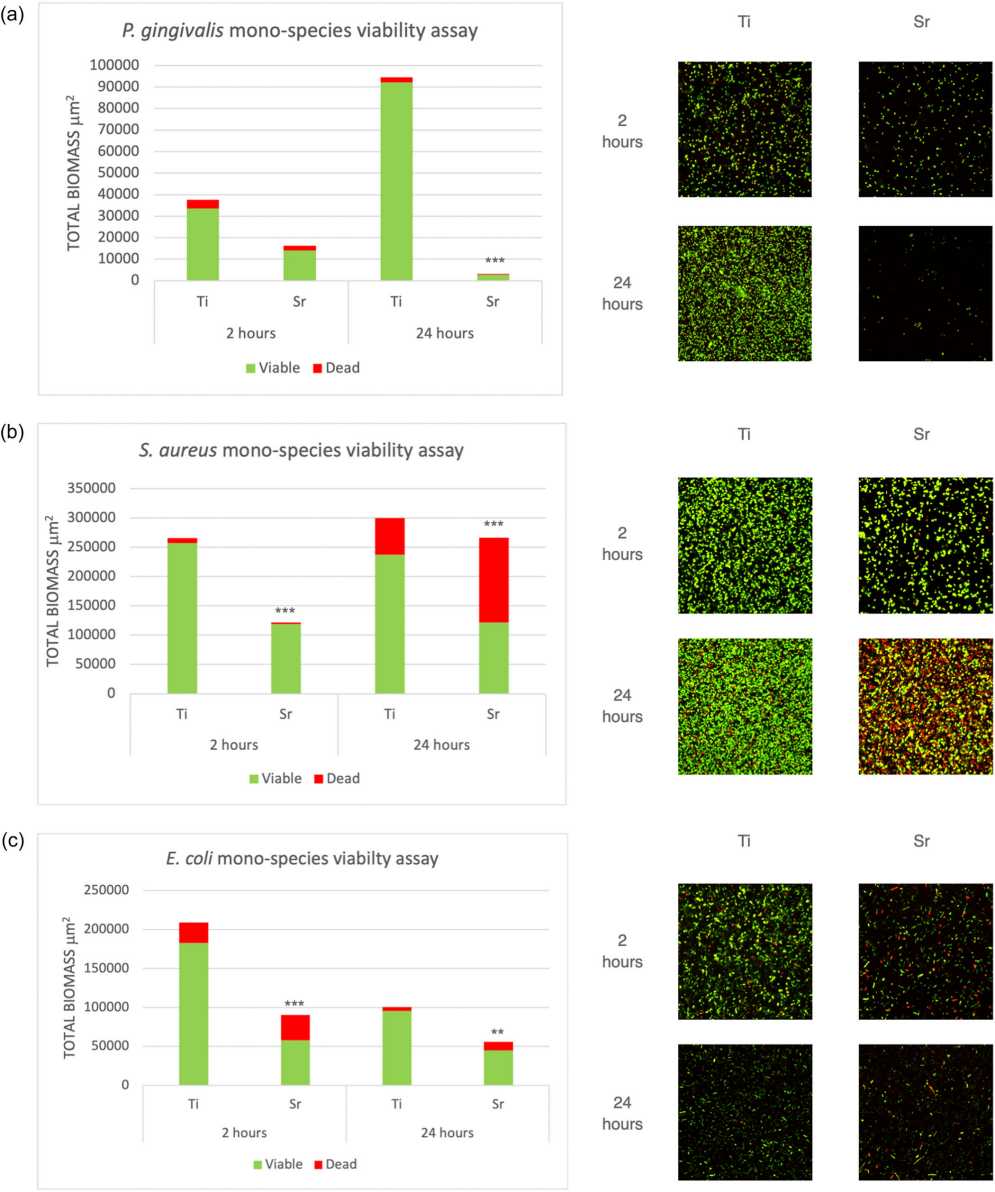
Bar graph showing the results of mono‐species viability assay showing total biomass, viable (green) and dead (red) cells, and CSLM images of both Sr‐functionalized and Ti control wafers tested against *Porphyromonas gingivalis* (a), *Staphylococcus aureus* (b), and *Escherichia coli* (c). **p < 0.01 compared to Ti control. ***p < 0.001 compared to Ti control.

**Figure 2:**
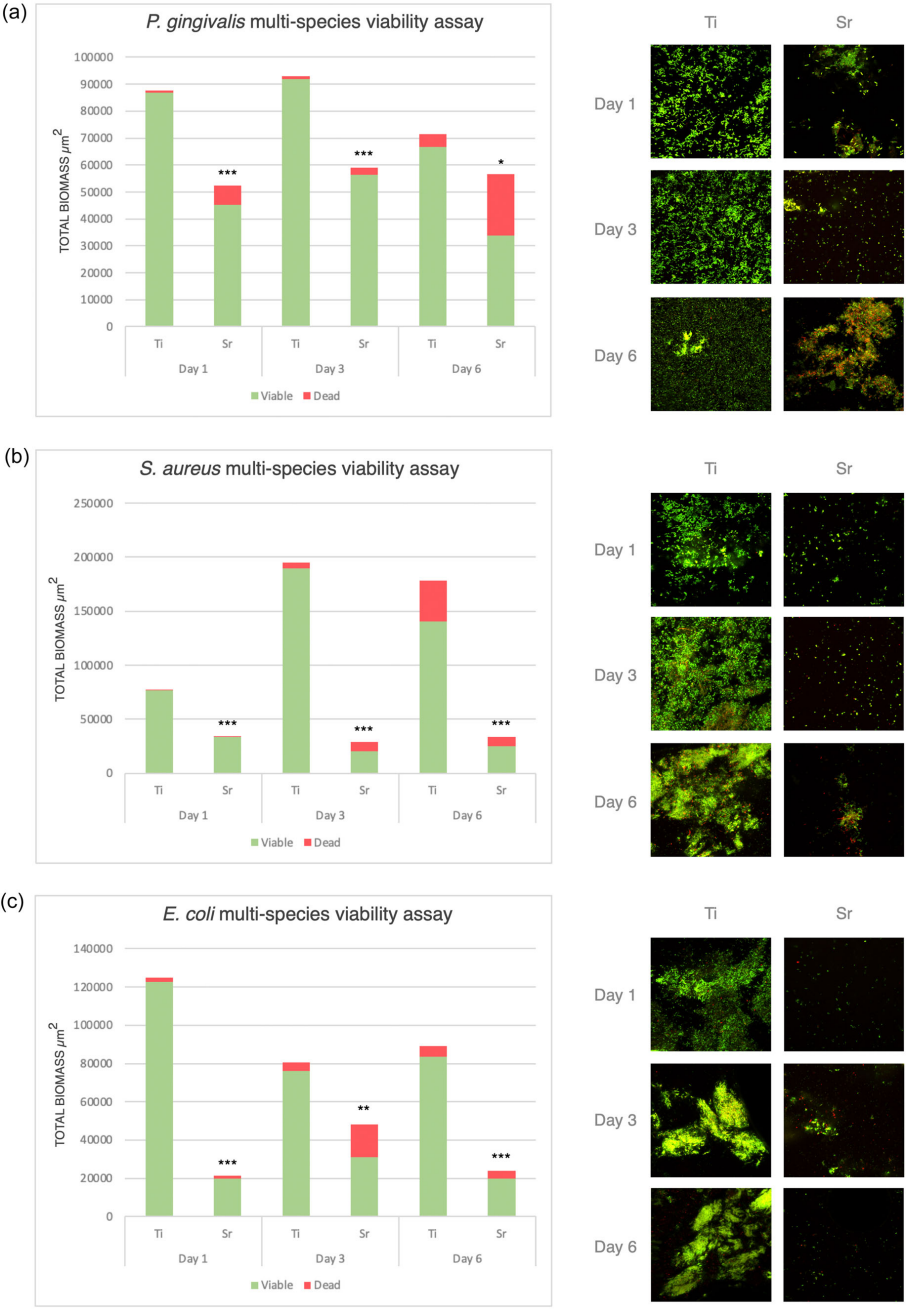
Bar graph showing the results of multispecies viability assay showing total biomass, viable (green) and dead (red) cells, and CSLM images of both Sr‐functionalized and Ti control wafers tested against *Porphyromonas gingivalis* multispecies (a), *Staphylococcus aureus* multispecies (b) and *Escherichia coli* multispecies consortia (c). *p < 0.05 compared to Ti control. **p < 0.01 compared to Ti control. ***p < 0.001 compared to Ti control.

For mono‐species biofilms, at 2 h, Sr‐functionalized wafers showed reduced numbers of viable cells compared to Ti control wafers for all bacteria tested, that is, *P. gingivalis*, *S. aureus* and *E. coli,* with the differences being statistically significant for *S. aureus* and *E. coli* (*p* < 0.001). Following 24 h of incubation, Sr‐functionalized wafers showed significantly less viable cells compared to Ti control wafers for all three species (Figure 1). For multispecies biofilms, following incubation for 1, 3, and 6 days, Sr‐functionalized wafers showed significantly less attached bacterial cells (i.e., total biomass) and significantly less viable cells for all different consortia mixtures, that is, *P. gingivalis*‐, *S. aureus*‐, and *E. coli*‐multispecies cultures compared with Ti control wafers (Figure 2).

### 3.2 Effect of Sr on Bacterial Growth in Multispecies Biofilms

**Figure 3:**
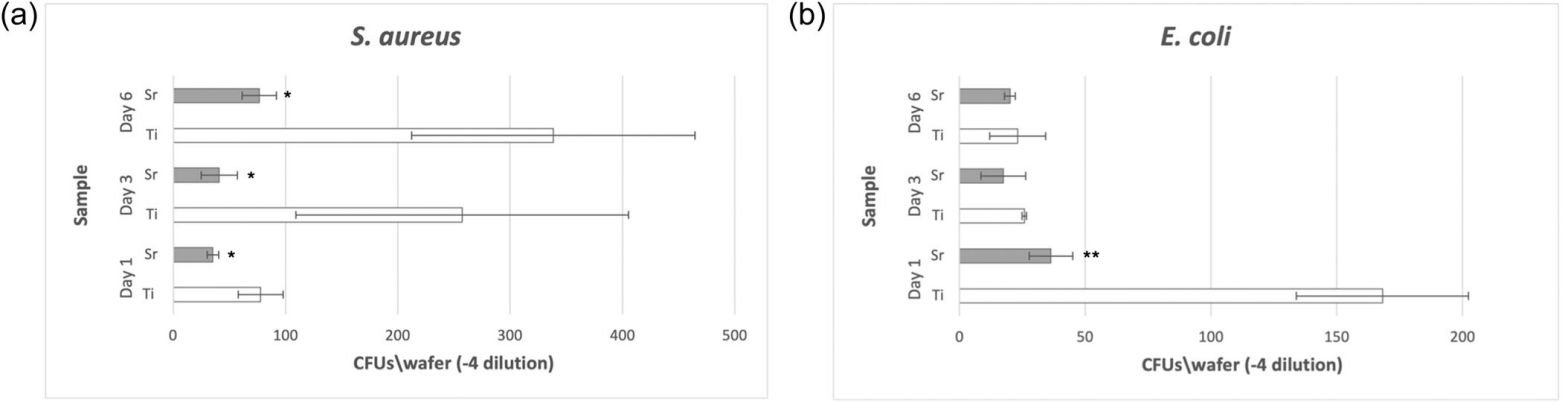
Multispecies plate counting method for *Staphylococcus aureus* and *Escherichia coli* multispecies test. Differences in number of *S. aureus* CFUs from multispecies consortium grown on 110‐plates (a) and *E. coli* CFUs grown on MacConkey plates (b) for each time point of evaluation comparing Sr‐functionalized and Ti control wafers. *p < 0.05 compared to Ti control. **p < 0.01 compared to Ti control.

The growth of *E. coli* and *S. aureus* bacteria in the multispecies biofilms on Sr‐functionalized and Ti control wafers was evaluated using serial dilution and plating on MacConkey and 110‐plates, for *E. coli* and *S. aureus,* respectively, after detaching the biofilms from the surfaces using vortexing. Following incubation of 2 days, 110‐plates inoculated from *S. aureus* multispecies showed a significantly lower number of CFU growth in Sr‐functionalized wafers compared to Ti control wafers at all time points of evaluation (Figure 3a). Similarly, a lower number of *E. coli* CFUs were associated with Sr‐functionalized wafers compared to Ti control wafers; however, the differences were statistically significant only at Day 1 (Figure 3b). The amount of *P. gingivalis* in the multispecies biofilms was evaluated using qPCR. *P. gingivalis* was detected only at Day 6, and only on Ti control wafers constituting 15% of the total biofilm, while it had remained below detection levels in all Sr‐functionalized wafers and at all time points of evaluation.

### 3.3 Effect of Sr on Proteolytic Activity

**Figure 4:**
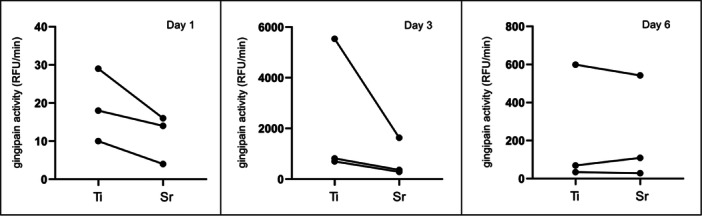
Proteolytic activity of the triplicate experiments of *Porphyromonas gingivalis* multispecies biofilm showing higher gingipains activity associated with Ti control wafers compared to Sr‐funtionalized wafers, except in only one experiment at Day 6.

Supernatants from biofilms grown on Sr‐functionalized wafers presented lower gingipain activity at all time points of evaluation, except from one single experiment at Day 6 (Figure 4). At Day 1, gingipain activity was low; however, it was always higher in Ti control wafers than in Sr‐functionalized wafers. The peak of gingipain activity was observed at Day 3; again, gingipain activity was in general rather higher in Ti control wafers compared to Sr‐functionalized wafers. A drop in gingipain activity was seen at Day 6, but again in two of the experiments, there was lower gingipain activity associated with Sr‐functionalized wafers (Figure 4).

## 4 Discussion

In the current study, Sr‐functionalized wafers presented a pronounced bacteriostatic and bactericidal potential against bacteria associated with peri‐implantitis, in both mono‐ and multispecies biofilms. Specifically, Sr‐functionalized wafers presented a lower number of attached and viable cells compared to Ti control wafers, basically for all bacterial species tested, from after a few hours of culturing and up to 6 days, the longest time point in this study. Furthermore, Sr‐functionalized wafers interfered with the growth and proteolytic activity of *P. ginigivalis*, a pathogen considered a keystone in the development of a dysbiotic biofilm (Hajishengallis and Lamont 2012) and commonly present in high numbers in peri‐implantitis lesions (Persson and Renvert 2014). Particularly, *P. gingivalis* remained below detection levels at all time points of evaluation, while gingipain activity was at all but one experiments lower in Sr‐functionalized samples, compared to Ti control wafers.

These results corroborate the findings of previous in vitro studies (O'Sullivan et al. 2010; Zhao et al. 2019; Zhou and Zhao 2016). For example, Zhou et al., using plate counting, reported antimicrobial rates of up to 40% against *S. aureus* and *E. coli,* of various types of coatings of Ti disks, when functionalized with Sr, within the first 24 h of testing (Zhou and Zhao 2016). Likewise, Zhao et al., using a similar method reported an antimicrobial rate of ca. 55% against *S. aureus* cultured on microstructured Ti specimens functionalized with Sr, after 24 h of testing. Furthermore, the finding herein, that Sr‐functionalized wafers exert bacteriostatic and bactericidal potential for up to 6 days, is also in agreement with the results of a previous in vitro study (O'Sullivan et al. 2010) showing a growth inhibition of *S. aureus* on Ti coupons functionalized with Sr‐substituted apatite of about 40% after 7 days. Thus, Sr appears to have a noticeable antimicrobial effect already at the early stages of bacterial attachment and continues as long as an adequate amount of Sr ions is released. Indeed, the limited antimicrobial effect of hydroxyapatite (HA) coatings functionalized with Sr was observed during the first 24 h, against *S. aureus*, *E. coli*, and *P. aeruginosa* mono‐species cultures, when low levels of Sr ions were released (Fielding et al. 2012; Geng et al. 2016). In fact, in both studies, a rapid cumulative increase in Sr ions concentration was observed after 24 h; however, no antimicrobial test was performed beyond the first 24 h (Fielding et al. 2012; Geng et al. 2016).

In this context, since Sr reacts with water to form Sr(OH)_2_, the observed antimicrobial effect of Sr‐functionalized wafers may be related to changes in the pH of the cultures (Jayasree et al. 2017). The magnetron sputtering process, used to functionalize the samples of the current study, deposits a thin coating on the wafer surface, with negligible (i.e., at the level of nm) surface roughness change; it has previously been shown that Sr‐functionalized turned Ti surfaces, produced with the same process as herein, release approximately 30 µg/cm^2^ Sr over 7 days (Offermanns, Andersen, et al. 2018). This means that within the culture volume of the current study, assuming the same ion release rate, the effective concentration of Sr^2+^ could reach ~0.1 mM with a corresponding of OH^−^ concentration of ~0.2 mM. Thus, considering Sr(OH)_2_ to be a strong base, one could calculate the pH of an aqueous solution to be ~10.3. Considering the experimental setup, it is likely that a concentration gradient would build up near the surface of the wafer, thus, yielding a region with a locally higher Sr(OH)_2_ concentration, that is, with an even higher pH. Most bacterial species prefer to grow in a pH around neutral and changes in the pH interfere with their ability to grow and negatively influence their viability. Indeed, the high pH as an explanation of the antibacterial effect of Sr has been demonstrated in a 6‐week in vitro study assessing Sr‐containing dental cement (Jayasree et al. 2017). Future studies should explore whether the antimicrobial effect of Sr observed herein is due to inducing a high alkaline pH.

As previously mentioned, Sr is particularly interesting because of its known potential to enhance bone formation by promoting osteoblast adhesion and proliferation, and also interfere with osteoclast function (López‐Valverde et al. 2019; Lu et al. 2021; Yamaguchi and Neale Weitzmann 2012). Indeed, systemic administration of Sr ranelate seems to somehow enhance peri‐implant bone quality and implant osseointegration in animals (Scardueli et al. 2018) and to enhance bone regeneration in grafted bone defects in healthy and osteoporotic rats (Mardas et al. 2021); while, loading a bovine bone substitute with Sr, significantly enhanced bone formation, compared with non‐loaded controls, when implanted in critical‐size calvarial defects in rats (Aroni et al. 2019). Furthermore, Sr functionalized Ti implants, produced with the same magnetron sputtering used herein, have exhibited enhanced osseointegration in several preclinical in vivo studies (Andersen et al. 2013; López‐Valverde et al. 2019; Offermanns, Andersen, et al. 2018).

In perspective, none of the previous studies assessing the antimicrobial potential of Sr coatings has used biofilms composed of multispecies bacteria. Thus, the present study used a multispecies model composed of four common oral microorganisms, combined with three different bacterial species that have been reported to be associated with peri‐implantitis. Nevertheless, it is estimated that more than 700 different bacterial species live in the oral cavity (Aas et al. 2005), with about 30% non‐culturable; obviously, the multispecies cultures herein, represent simply a negligible fraction of the oral biofilm. Furthermore, the oral cavity consists of a variety of tissues, and often a variety of restorative materials and surfaces, where bacterial attachment, biofilm composition, and function differ significantly. In this context, modern Ti dental implant surfaces show great variation and complexity in terms of topographic features, including roughness and microtopography, which are not represented by the smooth wafer surface used herein. Implant surface characteristics do not only have an impact on the biofilm behavior per se (e.g., modified/roughened implant surfaces are associated with increased risk for bacterial attachment and biofilm formation compared to turned/smooth ones) (Bermejo et al. 2019) but it may well be expected to modulate the release profile/amount of Sr, in connection/depending on the technology used to functionalize the Ti surface.

## 5 Conclusion

Sr‐functionalized Ti displayed promising antimicrobial potential, possessing bactericidal and bacteriostatic ability against bacteria associated with peri‐implantitis grown either as mono‐species or mixed in a multispecies consortium with different common oral microorganisms. Further testing Sr functionalization of clinically relevant implant surfaces seems warranted.

## Author Contributions

**Hatem Alshammari:** manuscript drafting, data generation, data interpretation. **Jessica Neilands:** study set‐up, data generation, supervision, manuscript reviewing. **Christian Sloth Jeppesen:** specimen preparation, manuscript reviewing. **Ole Zoffmann Andersen** and **Klaus Pagh Almtoft:** data interpretation, manuscript reviewing. **Andreas Stavropoulos:** study idea, funding, data interpretation, supervision, manuscript drafting.

## Conflicts of Interest

The authors declare no conflicts of interest.

## Data Availability

Data are available from the authors upon reasonable request.

## References

[cre2903-bib-0001] Aas, J. A. , B. J. Paster , L. N. Stokes , I. Olsen , and F. E. Dewhirst . 2005. “Defining the Normal Bacterial Flora of the Oral Cavity.” Journal of Clinical Microbiology 43, no. 11: 5721–5732. 10.1128/JCM.43.11.5721-5732.2005. 16272510 PMC1287824

[cre2903-bib-0002] Alshammari, H. , F. Bakitian , J. Neilands , O. Z. Andersen , and A. Stavropoulos . 2021. “Antimicrobial Properties of Strontium Functionalized Titanium Surfaces for Oral Applications, A Systematic Review.” Coatings 11, no. 7: 810.

[cre2903-bib-0003] Alshammari, H. , J. Neilands , G. Svensäter , and A. Stavropoulos . 2021. “Antimicrobial Potential of Strontium Hydroxide on Bacteria Associated With Peri‐Implantitis.” Antibiotics 10, no. 2: 150. 10.3390/antibiotics10020150. 33546189 PMC7913193

[cre2903-bib-0004] Andersen, O. Z. , V. Offermanns , M. Sillassen , et al. 2013. “Accelerated Bone Ingrowth by Local Delivery of Strontium From Surface Functionalized Titanium Implants.” Biomaterials 34, no. 24: 5883–5890. 10.1016/j.biomaterials.2013.04.031. 23672822

[cre2903-bib-0005] Aroni, M. A. T. , G. J. P. L. de Oliveira , L. C. Spolidório , et al. 2019. “Loading Deproteinized Bovine Bone With Strontium Enhances Bone Regeneration in Rat Calvarial Critical Size Defects.” Clinical Oral Investigations 23, no. 4: 1605–1614. 10.1007/s00784-018-2588-6. 30143902

[cre2903-bib-0006] Bermejo, P. , M. C. Sánchez , A. Llama‐Palacios , E. Figuero , D. Herrera , and M. Sanz Alonso . 2019. “Biofilm Formation on Dental Implants With Different Surface Micro‐Topography: An In Vitro Study.” Clinical Oral Implants Research 30, no. 8: 725–734. 10.1111/clr.13455. 31077449

[cre2903-bib-0007] Choi, S. H. , Y. S. Jang , J. H. Jang , T. S. Bae , S. J. Lee , and M. H. Lee . 2019. “Enhanced Antibacterial Activity of Titanium by Surface Modification With Polydopamine and Silver for Dental Implant Application.” Journal of Applied Biomaterials & Functional Materials 17, no. 3: 228080001984706. 10.1177/2280800019847067. 31530071

[cre2903-bib-0008] Chouirfa, H. , H. Bouloussa , V. Migonney , and C. Falentin‐Daudré . 2019. “Review of Titanium Surface Modification Techniques and Coatings for Antibacterial Applications.” Acta Biomaterialia 83: 37–54. 10.1016/j.actbio.2018.10.036. 30541702

[cre2903-bib-0009] Derks, J. , D. Schaller , J. Håkansson , J. L. Wennström , C. Tomasi , and T. Berglundh . 2016. “Effectiveness of Implant Therapy Analyzed in a Swedish Population: Prevalence of Peri‐Implantitis.” Journal of dental research 95, no. 1: 43–49. 10.1177/0022034515608832. 26701919

[cre2903-bib-0010] Donos, N. , E. Calciolari , M. Ghuman , M. Baccini , V. Sousa , and L. Nibali . 2023. “The Efficacy of Bone Reconstructive Therapies in the Management of Peri‐Implantitis. A Systematic Review and Meta‐Analysis.” Supplement, Journal of Clinical Periodontology 50, no. 26: 285–316. 10.1111/jcpe.13775. 36635029

[cre2903-bib-0011] Fielding, G. A. , M. Roy , A. Bandyopadhyay , and S. Bose . 2012. “Antibacterial and Biological Characteristics of Silver Containing and Strontium Doped Plasma Sprayed Hydroxyapatite Coatings.” Acta Biomaterialia 8, no. 8: 3144–3152. 10.1016/j.actbio.2012.04.004. 22487928 PMC3393112

[cre2903-bib-0012] Geng, Z. , Z. Cui , Z. Li , et al. 2016. “Strontium Incorporation to Optimize the Antibacterial and Biological Characteristics of Silver‐Substituted Hydroxyapatite Coating.” Materials Science and Engineering: C 58: 467–477. 10.1016/j.msec.2015.08.061. 26478334

[cre2903-bib-0013] Grischke, J. , J. Eberhard , and M. Stiesch . 2016. “Antimicrobial Dental Implant Functionalization Strategies—A Systematic Review.” Dental Materials Journal 35, no. 4: 545–558. 10.4012/dmj.2015-314. 27477219

[cre2903-bib-0014] Hadrup, N. , A. K. Sharma , and K. Loeschner . 2018. “Toxicity of Silver Ions, Metallic Silver, and Silver Nanoparticle Materials After In Vivo Dermal and Mucosal Surface Exposure: A Review.” Regulatory Toxicology and Pharmacology 98: 257–267. 30125612 10.1016/j.yrtph.2018.08.007

[cre2903-bib-0015] Hajishengallis, G. , and R. J. Lamont . 2012. “Beyond the Red Complex and Into More Complexity: The Polymicrobial Synergy and Dysbiosis (PSD) Model of Periodontal Disease Etiology.” Molecular Oral Microbiology 27, no. 6: 409–419. 10.1111/j.2041-1014.2012.00663.x. 23134607 PMC3653317

[cre2903-bib-0016] Heitz‐Mayfield, L. J. A. , and G. E. Salvi . 2018. “Peri‐Implant Mucositis.” Supplement, Journal of Clinical Periodontology 45: S237–S245. 29926488 10.1111/jcpe.12953

[cre2903-bib-0017] Jayasree, R. , T. S. S. Kumar , S. Mahalaxmi , S. Abburi , Y. Rubaiya , and M. Doble . 2017. “Dentin Remineralizing Ability and Enhanced Antibacterial Activity of Strontium and Hydroxyl Ion Co‐Releasing Radiopaque Hydroxyapatite Cement.” Journal of Materials Science: Materials in Medicine 28, no. 6: 95. 10.1007/s10856-017-5903-x. 28502026

[cre2903-bib-0018] Jemt, T. 2017. “A Retro‐Prospective Effectiveness Study on 3448 Implant Operations at One Referral Clinic: A Multifactorial Analysis. Part I: Clinical Factors Associated to Early Implant Failures.” Clinical Implant Dentistry and Related Research 19: 980–988. 28884953 10.1111/cid.12539

[cre2903-bib-0019] Karlsson, K. , A. Trullenque‐Eriksson , C. Tomasi , and J. Derks . 2023. “Efficacy of Access Flap and Pocket Elimination Procedures in the Management of Peri‐Implantitis: A Systematic Review and Meta‐Analysis.” Supplement, Journal of Clinical Periodontology 50, no. S26: 244–284. 10.1111/jcpe.13732. 36217689

[cre2903-bib-0020] Klinge, B. , A. Klinge , K. Bertl , and A. Stavropoulos . 2018. “Peri‐Implant Diseases.” Supplement, European Journal of Oral Sciences 126, no. S1: 88–94. 10.1111/eos.12529. 30178555

[cre2903-bib-0021] Lin, G. , S. Ye , F. Liu , and F. He . 2018. “A Retrospective Study of 30,959 Implants: Risk Factors Associated With Early and Late Implant Loss.” Journal of Clinical Periodontology 45: 733–743. 29608788 10.1111/jcpe.12898

[cre2903-bib-0022] López‐Valverde, N. , J. Muriel‐Fernández , R. Gómez de Diego , J. Ramírez , and A. López‐Valverde . 2019. “Effect of Strontium‐Coated Titanium Implants on Osseointegration in Animal Models: A Literature Systematic Review.” The International Journal of Oral & Maxillofacial Implants 34, no. 34: 1389–1396. 10.11607/jomi.7827. 31711080

[cre2903-bib-0023] Lu, W. , Y. Zhou , H. Yang , Z. Cheng , and F. He . 2021. “Efficacy of Strontium Supplementation on Implant Osseointegration Under Osteoporotic Conditions: A Systematic Review.” The Journal of Prosthetic Dentistry 128: 341–349. 10.1016/j.prosdent.2020.12.031. 33589234

[cre2903-bib-0024] Mardas, N. , X. Dereka , A. Stavropoulos , M. Patel , and N. Donos . 2021. “The Role of Strontium Ranelate and Guided Bone Regeneration in Osteoporotic and Healthy Conditions.” Journal of Periodontal Research 56, no. 2: 330–338. 10.1111/jre.12825. 33368312

[cre2903-bib-0025] Masamoto, K. , S. Fujibayashi , S. Yamaguchi , et al. 2021. “Bioactivity and Antibacterial Activity of Strontium and Silver Ion Releasing Titanium.” Journal of Biomedical Materials Research Part B: Applied Biomaterials 109, no. 2: 238–245. 10.1002/jbm.b.34695. 32767436

[cre2903-bib-0026] Neilands, J. , F. J. Bikker , and B. Kinnby . 2016. “PAI‐2/Serpin B2 Inhibits Proteolytic Activity in a *P. gingivalis*‐Dominated Multispecies Bacterial Consortium.” Archives of Oral Biology 70: 1–8. 10.1016/j.archoralbio.2016.05.016. 27295389

[cre2903-bib-0027] O'Donnell, S. , A. Cranney , G. A. Wells , J. D. Adachi , and J. Y. Reginster . 2006. “Strontium Ranelate for Preventing and Treating Postmenopausal Osteoporosis.” Cochrane Database of Systematic Reviews no. 3: CD005326. 10.1002/14651858.CD005326.pub2. 16856092

[cre2903-bib-0028] O'Sullivan, C. , P. O'Hare , N. D. O'Leary , et al. 2010. “Deposition of Substituted Apatites With Anticolonizing Properties Onto Titanium Surfaces Using a Novel Blasting Process.” Journal of Biomedical Materials Research Part B: Applied Biomaterials 95, no. 1: 141–149. 10.1002/jbm.b.31694. 20737556

[cre2903-bib-0029] Offermanns, V. , O. Z. Andersen , M. Sillassen , et al. 2018. “A Comparative In Vivo Study of Strontium‐Functionalized and Slactive Implant Surfaces in Early Bone Healing.” International Journal of Nanomedicine 13: 2189–2197. 10.2147/IJN.S161061. 29692613 PMC5903483

[cre2903-bib-0030] Offermanns, V. , O. Steinmassl , O. Z. Andersen , et al. 2018. “Comparing the Effect of Strontium‐Functionalized and Fluoride‐Modified Surfaces on Early Osseointegration.” Journal of Periodontology 89, no. 8: 940–948. 10.1002/JPER.17-0680. 29697142

[cre2903-bib-0031] Pandolfi, A. , F. Rinaldo , D. Pasqualotto , F. Sorrentino , G. La Torre , and F. Guerra . 2019. “A Retrospective Cohort Study on Peri‐Implant Complications in Implants up to 10 Years of Functional Loading in Periodontally Compromised Patients.” Journal of Periodontology 91: 995–1002. 10.1002/JPER.18-0715. 31860130

[cre2903-bib-0032] Persson, G. R. , and S. Renvert . 2014. “Cluster of Bacteria Associated With Peri‐Implantitis.” Clinical Implant Dentistry and Related Research 16, no. 6: 783–793. 10.1111/cid.12052. 23527870

[cre2903-bib-0033] Scardueli, C. R. , C. Bizelli‐Silveira , R. A. C. Marcantonio , E. Marcantonio , A. Stavropoulos , and R. Spin‐Neto . 2018. “Systemic Administration of Strontium Ranelate to Enhance the Osseointegration of Implants: Systematic Review of Animal Studies.” International Journal of Implant Dentistry 4, no. 1: 21. 10.1186/s40729-018-0132-8. 30014305 PMC6047953

[cre2903-bib-0034] Stavropoulos, A. , K. Bertl , S. Eren , and K. Gotfredsen . 2019. “Mechanical and Biological Complications After Implantoplasty—A Systematic Review.” Clinical Oral Implants Research 30, no. 9: 833–848. 10.1111/clr.13499. 31254417

[cre2903-bib-0035] Stevenson, M. , S. Davis , M. Lloyd‐Jones , and C. Beverley . 2007. “The Clinical Effectiveness and Cost‐Effectiveness of Strontium Ranelate for the Prevention of Osteoporotic Fragility Fractures in Postmenopausal Women.” Health Technology Assessment 11, no. 4: 1–134. 10.3310/hta11040. 17280622

[cre2903-bib-0036] Yamaguchi, M. , and M. Neale Weitzmann . 2012. “The Intact Strontium Ranelate Complex Stimulates Osteoblastogenesis and Suppresses Osteoclastogenesis by Antagonizing NF‐ΚB Activation.” Molecular and Cellular Biochemistry 359, no. 1–2: 399–407. 10.1007/s11010-011-1034-8. 21874315

[cre2903-bib-0037] Zhang, X. , H. Wu , Z. Geng , et al. 2014. “Microstructure and Cytotoxicity Evaluation of Duplex‐Treated Silver‐Containing Antibacterial TiO_2_ Coatings.” Materials Science and Engineering: C 45: 402–410. 10.1016/j.msec.2014.07.002. 25491845

[cre2903-bib-0038] Zhao, Q. , L. Yi , L. Jiang , Y. Ma , H. Lin , and J. Dong . 2019. “Surface Functionalization of Titanium With Zinc/Strontium‐Doped Titanium Dioxide Microporous Coating via Microarc Oxidation.” Nanomedicine: Nanotechnology, Biology and Medicine 16: 149–161. 10.1016/j.nano.2018.12.006. 30594657

[cre2903-bib-0039] Zhou, J. , and L. Zhao . 2016. “Multifunction Sr, Co and F Co‐Doped Microporous Coating on Titanium of Antibacterial, Angiogenic and Osteogenic Activities.” Scientific Reports 6: 29069. 10.1038/srep29069. 27353337 PMC4926257

